# NiCo alloy nanoparticles electrodeposited on an electrochemically reduced nitrogen-doped graphene oxide/carbon-ceramic electrode: a low cost electrocatalyst towards methanol and ethanol oxidation

**DOI:** 10.1039/c9ra06290d

**Published:** 2019-10-23

**Authors:** Kaveh Rahmani, Biuck Habibi

**Affiliations:** Electroanalytical Chemistry Laboratory, Department of Chemistry, Faculty of Sciences, Azarbaijan Shahid Madani University Tabriz 53714-161 Iran B.Habibi@azaruniv.ac.ir +98 41 34327541 +98 41 31452079

## Abstract

In this work, nickel–cobalt alloy nanoparticles were electrodeposited on/in an electrochemically reduced nitrogen-doped graphene oxide (ErN-GO)/carbon-ceramic electrode (CCE) and the resulting nanocomposite (NiCo/ErN-GO/CCE) was evaluated as a low cost electrocatalyst for methanol and ethanol electrooxidation. Field-emission scanning electron microscopy coupled with energy dispersive X-ray spectroscopy, X-ray diffraction, and Fourier-transform infrared spectroscopy were used for the physical characterization of the electrocatalyst. To study the electrochemical behavior and electrocatalytic activity of the prepared electrocatalyst towards the oxidation of methanol and ethanol in alkaline media, cyclic voltammetry, chronoamperometry and electrochemical impedance spectroscopy were utilized. Electrochemical investigation of the introduced electrocatalysts (NiCo alloy and Ni nanoparticles alone electrodeposited on/in different substrates) indicated that NiCo/ErN-GO/CCE has highest activity and stability towards methanol (*J*_p_ = 88.04 mA cm^−2^) and ethanol (*J*_p_ = 64.23 mA cm^−2^) electrooxidation, which highlights its potential use as an anodic material in direct alcohol fuel cells.

## Abstract 2

NiCo alloy nanoparticles on the electrochemically reduced nitrogen-doped graphene oxide/carbon-ceramic electrode: a low cost electrocatalyst towards methanol and ethanol oxidation.

## Graphical abstract

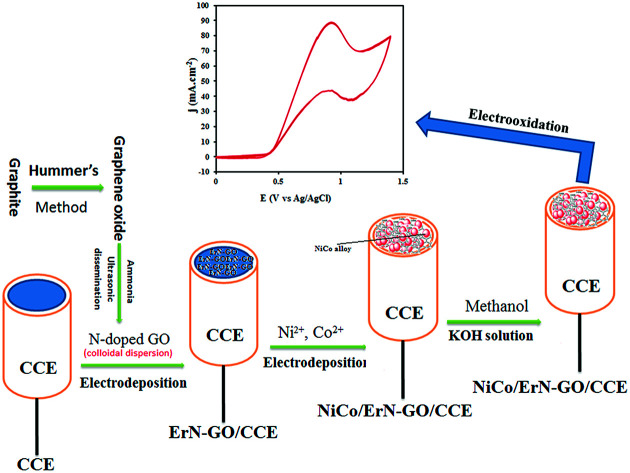

## 1. Introduction

Direct alcohol fuel cells (DAFCs) have several advantages in comparison with other types of fuel cells: high energy density, simpler system design, and no need for high pressures or temperatures.^1–3^ Therefore, DAFCs have many stationary and portable applications in different fields, since they have low noise and thermal signatures, no toxic effluent, and high theoretical and higher specific energy density.^1,3,4^ Methanol and ethanol are the most common fuels used in DAFCs, because of their many benefits such as low cost, easy handling, transportation, and storage and can be obtained in great quantity from renewable resources.^5–7^ However, even though platinum (Pt) is the best electrocatalyst for the electrooxidation of alcohols it is a high cost material with low abundance on our planet.^8^ On the other hand, beside the sluggish electrooxidation kinetics of methanol and ethanol on Pt and Pt-based electrocatalysts, their reactions produce byproducts and intermediates that adsorb onto the Pt particle surfaces and decrease their electrocatalytic activity; causing poisoning.^9^ Consequently, to achieve lower or no Pt uses and higher poison tolerance, numerous attempts have been made to provide non-Pt-based electrocatalysts.^10–12^ Therefore, development of new, low cost and non-Pt-based electrocatalysts is one of the main goals of the scientific communities in the field of fuel cells and energy conversations. One of the first non-precious metals proposed to be used for alcohol electrooxidation is nickel (Ni).^13–15^ It is well known that the Ni has advantages as an electrocatalyst because of its surface characteristics and demonstrated electrocatalytic activity towards methanol and ethanol oxidation.^16–18^ However, smooth Ni shows poor electrocatalytic activity, thus, to improve the electrocatalytic activity towards alcohol oxidation, its nanosize structures with high dispersion rate are necessary.^19^ Electrocatalytic activity of Ni-based materials also may be improved by incorporating other chemical elements with low oxidation potentials that accelerates the Ni^2+^/Ni^3+^ redox reaction.^20^ In this case, Ni-based electrocatalysts such as NiCu, NiCr, NiMn, *etc.* were prepared and investigated.^21–23^ Although, cobalt (Co) has high catalytic properties for some chemical reactions, it unused as the main electrocatalyst material for electrooxidation of methanol and ethanol due to its low performance.^24^ Since Ni and Co are neighbor in the periodic table, they have similar crystal structure, and their lattice parameters are very close, so Ni and Co can form solid solution alloys.^25,26^ The enhanced electrocatalytic activities of these materials have been explained by the well-known bifunctional mechanism, where the Co metal promotes the adsorption of OH^−^ at low potentials that facilitates the formation of the NiOOH active sites.^27,28^ Furthermore, it has been observed that the second or third element in the compound might improve the stability of Ni-based electrocatalysts.^27,29^

On the other hand, development of an active support material such as graphene, graphene oxide (GO) and reduced graphene oxide (rGO), emerges as efficient materials due to their low cost, mechanical strength, large specific surface area, enhances conductivity and flexibility towards further modifications and enhance stability of metal nanoparticles.^30–32^ Also, doping of graphene materials with heteroatoms such as nitrogen (N), sulfur (S) or phosphor (P), can helps to modify the electronic structure and physicochemical characteristics of these nanomaterials.^33,34^ Among them, N has received considerable attention because of its homogeneous size compared to carbon since it can donate the extra electron easily to the carbon substrates. In the presence of N atoms, there is less chance for restacking, so metal nanoparticles deposited in the GO layers homogenously. Furthermore, N-doped GO nanomaterial has been identified as a superior substrate to graft active nanoparticles for various applications such as a highly conductive matrix for producing strong connection between the electrode–electrolyte interface that accelerate the electron transfer rate of the carbon materials during electrocatalytic processes.^35^

In this work, nickel–cobalt (NiCo) alloy nanoparticles are electrodeposited on/in the electrochemically reduced nitrogen-doped graphene oxide (ErN-GO)/carbon-ceramic electrode (CCE) by the galvanostatic method to preparation of the NiCo/ErN-GO/CCE electrocatalyst. Field-emission scanning electron microscopy coupled with an energy dispersive X-ray spectroscopy, X-ray diffraction, and Fourier-transform infrared spectroscopy are used to the physical characterization of the electrocatalyst. NiCo/ErN-GO/CCE is used as a low cost electrocatalyst for electrooxidation of methanol and ethanol in alkaline media. Electrochemical investigations of the introduced electrocatalysts (NiCo and alone Ni nanoparticles electrodeposited on different substrates) indicate that the NiCo/ErN-GO/CCE has the highest activity and stability towards methanol and ethanol electrooxidation in alkaline media.

## 2. Experimental

### 2.1. Chemicals

Graphite, methyltrimethoxysilane (MTMOS), methanol, ethanol, KOH, NaH_2_PO_4_·12H_2_O, KH_2_PO_4_, CoCl_2_·5H_2_O, NiCl_2_·6H_2_O, KMnO_4_, NH_4_Cl, H_3_BO_3_, H_2_O_2_, NH_3_, HCl, and H_2_SO_4_ were purchased from Merck Company. All chemicals were of analytical grade and were used without further purification. All solutions were prepared with double-distilled water.

### 2.2. Instrumentation

All electrochemical measurements were performed using a SAMA 500 (potentiostat/galvanostat) equipped with a USB electrochemical interface. A conventional three electrodes cell was used for electrochemical experiments at room temperature. An Ag/AgCl and a platinum wire (AZAR ELECTRODE CO.) were used as the reference and auxiliary electrode, respectively. Fourier transform infrared spectroscopy (FTIR) (PerkinElmer, Spectrum 100) was employed for the analysis the nature of chemical bonds in terms of the functional group of the synthesized GO and N-GO. Field emission scanning electron microscopy (FESEM), energy-dispersive X-ray spectroscopy (EDX) and EDX-mapping were performed on a TESCAN, MIRA III model instrument. X-ray Diffraction (XRD) of the nanomaterials was studied using a Brucker AXF (D8 Advance) X-ray powder diffractometer with a Cu Kα radiation source (*λ* = 0.154056 nm) generated at 40 kV and 35 mA. Furthermore, electrochemical impedance spectroscopy (EIS) measurements were carried out in the frequency range of 0.1 Hz to 10 kHz under amplitude of 10 mV and constant bias potential 0.6 V by AUTOLAB potentiostat/galvanostat PGSTAT100 equipped with EIS.

### 2.3. The procedure of CCE, ErGO/CCE and ErN-GO/CCE preparation

For fabrication of the CCE, the sol–gel process was used according to our previous work.^36^ The modified Hummer's method was used to produce GO from natural graphite.^37^ For electrochemical preparation of the ErGO on CCE, ErGO/CCE, the polished CCE was immersed into the GO colloid (a colloidal dispersion of 1.0 mg ml^−1^ GO) and its potential was swept from −1.5 to 0.5 V for 10 cycles at a scan rate of 5 mV s^−1^.^38^ Nitrogen-doped graphene oxide (N-GO) was prepared by a chemical process through the ultrasonic dissemination of GO in ammonia solution (25% w/w) at 5–7 °C.^39^ Normally, 0.4 g of GO powder was mixed with 100 ml of ammonia solution. The resulting dispersion was exposed to ultrasonic bath at the mentioned temperature for about 5 hours. The resulted precipitate was gathered by centrifugation, washed with distilled water three times, and then dried at 60 °C for 24 h. To prepare ErN-GO/CCE, firstly a sufficient amount of N-GO was dissolved in a solution of 0.07 M phosphate buffer solution (PBS) to provide a 1 mg ml^−1^ N-GO colloidal dispersion. Subsequently, the polished CCE was placed in the N-GO colloidal dispersion, and cyclic voltammetry in the range of −1.5 to 0.5 V *vs.* Ag/AgCl at a scan rate of 5 mV s^−1^ (10 cycles) was used to prepare of the ErN-GO/CCE. The obtained electrodes then rinsed with distilled water and dried at room temperature overnight.

### 2.4. Electrodeposition of NiCo and alone Ni nanoparticles on different substrates

For electrodeposition of NiCo alloy nanoparticles on/in the ErN-GO/CCE, the galvanostatic method^40–42^ was used in a solution containing proper amount of NiCl_2_·6H_2_O, CoCl_2_·5H_2_O (nickel and cobalt molar ratio of 3 : 1) and 1 M NH_4_Cl + 0.7 M NH_4_OH + 2 M boric acid (pH adjusted at 6) as deposition electrolyte at the current density of 60 mA cm^−2^ for 100 s. Boric acid improves the required current density for electrodeposition process and also advances the appearance characteristics of the coating and decreases the coating fragility.^43,44^ Then the modified electrode was rinsed with deionized water and dried at room temperature. To conduct further investigations of the N-doping effect, ErN-GO, efficiency and Co presence in physical and electrochemical properties of prepared electrocatalysts, Ni/CCE, Ni/ErN-GO/CCE, NiCo/CCE, and NiCo/ErGO/CCE were prepared according to the above method. For electrodeposition of alone Ni nanoparticles on the CCE and ErN-GO/CCE, the same method (galvanostatic) was used in the presence of only Ni ions. The geometrical area of the CCE electrode was used in measurements of current density. All electrocatalytic studies were done at room temperature.

## 3. Results and discussion

### 3.1. Physical characterization

**Fig. 1:**
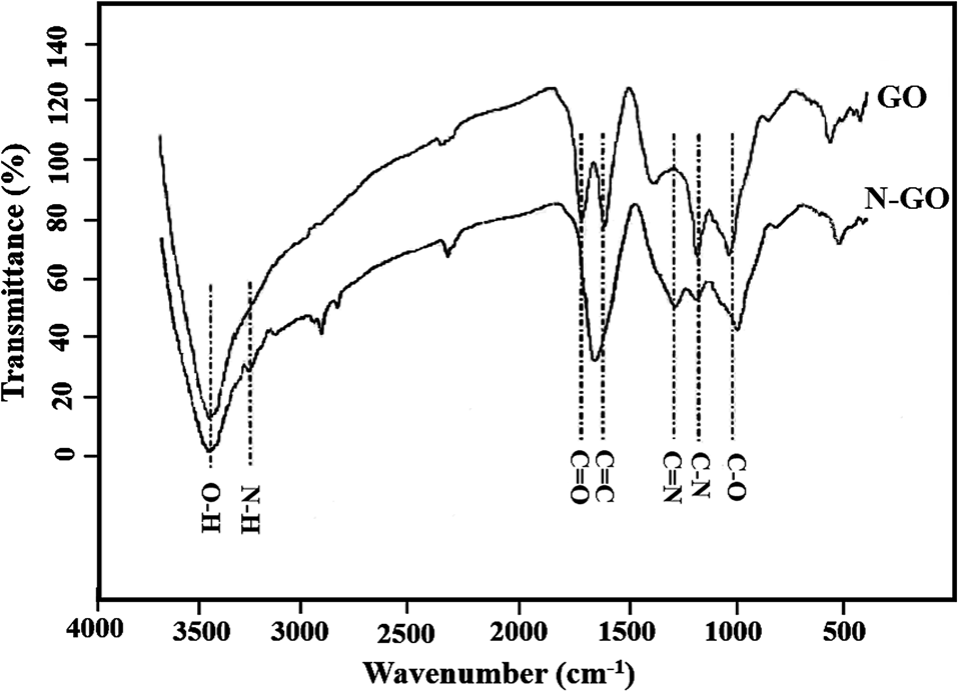
FTIR spectra of GO and N-GO.

Nitrogen-doped graphene oxide (N-GO) is initially prepared by using NH_4_OH as the nitrogen source. Fig. 1 shows the FTIR spectrum of the GO and N-GO. As can be seen from the FTIR of the GO and N-GO, the peak at 3440 cm^−1^ is related to O–H stretching vibration of water molecules adsorbed on both GO and N-GO. The characteristic peaks of GO such as the absorption peaks included 1010, 1610 and 1725 cm^−1^ were observed for stretching vibrational modes of C–O (alkoxy), C–O–C (epoxy), C=O and C=C (carbonyl) stretching vibrations at 1052.30, 1222.42, 1704.66 and 1590 cm^−1^ respectively and their relative intensities changed after nitrogen doping.^45,46^ After N-doping significant decrease in intensity of C=O peak was observed and approximately merged with C=C peak. Also, the peaks at about 1380 and 1210 cm^−1^ were appeared after N doping indicating the presence of C=N and C–N, respectively; which successful preparation of the N-GO can be proven.^47^ Another indication of successful incorporation of N in GO was observed at about 3250 cm^−1^ which attributes to N–H bonds in N-GO.

**Fig. 2:**
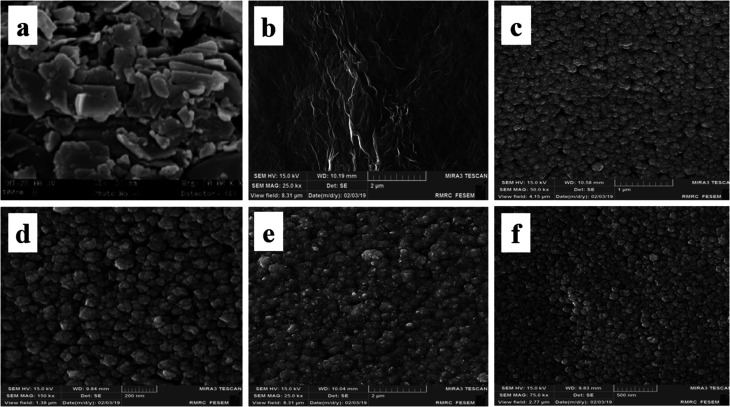
FESEM images of (a) CCE, (b) ErN-GO/CCE, (c) Ni/CCE, (d) Ni/ErN-GO/CCE, (e) NiCo/CCE and (f) NiCo/ErN-GO/CCE.

In order to surface characterization of the prepared electrocatalysts, their surface morphology is investigated by FESEM and associated results are shown in Fig. 2: CCE (image a), ErN-GO/CCE (image b), Ni/CCE (image c), Ni/ErN-GO/CCE (image d), NiCo/CCE (image e) and NiCo/ErN-GO/CCE (image f). As shown in Fig. 2 (image a), bare CCE (polished with emery paper grade 1500) has a porous and flaky surface. Fig. 2 (image b) shows the CCE surface after electrodeposition of N-GO (ErN-GO) by cyclic voltammetry. As seen in Fig. 2 (image b), rough irregular shaped grained structures on CCE surface formed which approves the synthesis of ErN-GO. Fig. 2 (image c) shows the FESEM micrographs of the Ni/CCE that reveals Ni nanoparticles uniformly dispersed over the CCE. Image d in Fig. 2 shows the FESEM morphology of the Ni/ErN-GO/CCE. In comparison with Ni/CCE, Ni nanoparticles electrodeposited more uniformly on the surface of ErN-GO/CCE due to the wrinkle-like and layered structure of the ErN-GO nanosheets with random directions. Fig. 2 (image e) shows the NiCo nanoparticles electrodeposited on the surface of CCE. It can be seen that NiCo with nano-sized particles have a uniform distributed structure and homogeneously dispersed in size with spherical structure. The FESEM image of NiCo/ErN-GO/CCE, image f in Fig. 2, shows surrounded ErN-GO layers with supported nano-sized NiCo alloy particles. Therefore, the surface coarseness of the electrodeposited film is highly improved by the presence of the ErN-GO. Additionally, as shown in Fig. 2 (image f) NiCo/ErN-GO/CCE has smaller average particle size (24.60 nm) in comparison with NiCo/CCE (36.80 nm) due to the uniform distribution of NiCo nanoparticles on layered structure of ErN-GO/CCE surface. This result corresponded with the enhancement of electrochemically active surface area due to increasing of the electrocatalytic performance of the prepared electrocatalyst. In other words, this superior morphology supplied more electrochemical active sites, which results in faster ionic transport in the electrode and electrolyte interface and improves the electrochemical activity of the resulting nanocomposite electrode.

**Fig. 3:**
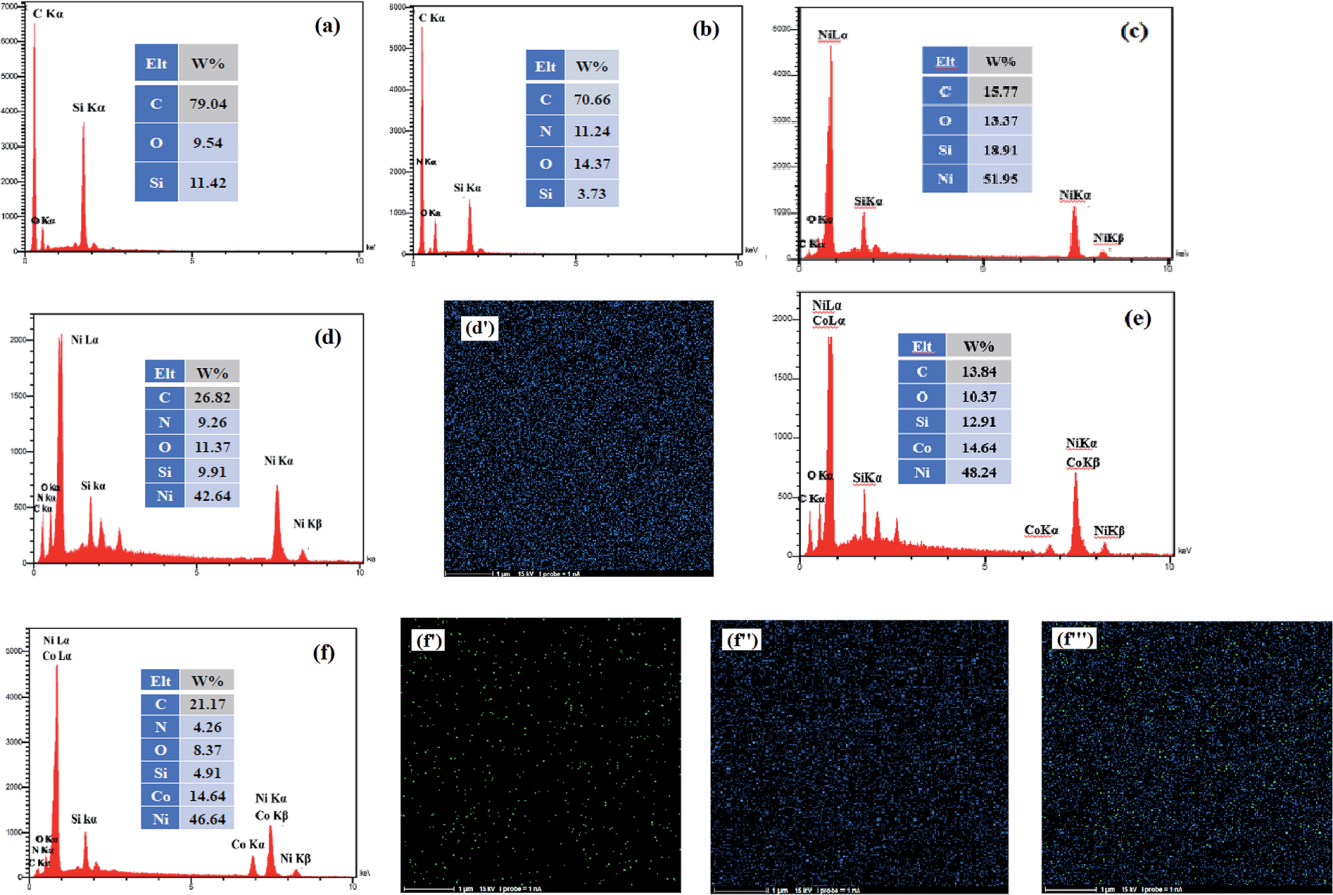
EDX spectra of (a) CCE, (b) ErN-GO/CCE, (c) Ni/CCE, (d) Ni/ErN-GO/CCE, (e) NiCo/CCE and (f) NiCo/ErN-GO/CCE. Insets are the surface chemical composition of the same electrodes. Elemental maps of Ni atoms in Ni/ErN-GO/CCE (d′) and Co atoms (f′), Ni atoms (f′′) and Ni, Co atoms (f′′′) in NiCo/ErN-GO/CCE.

EDX measurement is used to analysis of the surface chemical composition of the prepared electrocatalysts. The EDX spectra obtained for the bare CCE (a), ErN-GO/CCE (b), Ni/CCE (c), Ni/ErN-GO/CCE (d), NiCo/CCE (e) and NiCo/ErN-GO/CCE (f) are shown in Fig. 3. Inset in each spectrum shows the surface chemical composition of the same electrode. The presence of N peak in EDX spectrum of ErN-GO/CCE indicates that the N atoms loaded in the GO layers and N-GO nanosheets successfully electrodeposited on CCE (spectrum b).^48^ On the other hand, it is obvious that Ni and NiCo elements are present on the CCE (spectra of c and e) and also on the ErN-GO/CCE (spectra of d and f), which prove the successful electrodeposition of the Ni and NiCo nanoparticles on the surface of the substrates. Furthermore, element-mapping analysis is employed to analyze the elemental morphology and composition distribution of the Ni and NiCo in the prepared electrocatalysts and the obtained results are also shown in Fig. 3; Ni atoms in the Ni/ErN-GO/CCE (map d′) and NiCo/ERN-GO/CCE [maps of f′ (Co atoms), f′′ (Ni atoms) and f′′′ (Co and Ni atoms)]. It can be seen that the Ni and NiCo nanoparticles have been homogenously distributed on the ErN-GO/CCE substrate. From the maps of f′, f′′ and f′′′, it is observe that the two elements of Ni and Co are accompanied and uniformly distributed with concentrations distribution corresponding to the ratio of NiCo (3 : 1) on the ErN-GO/CCE, suggesting the well-homogeneous structure of NiCo alloy nanoparticles.

**Fig. 4:**
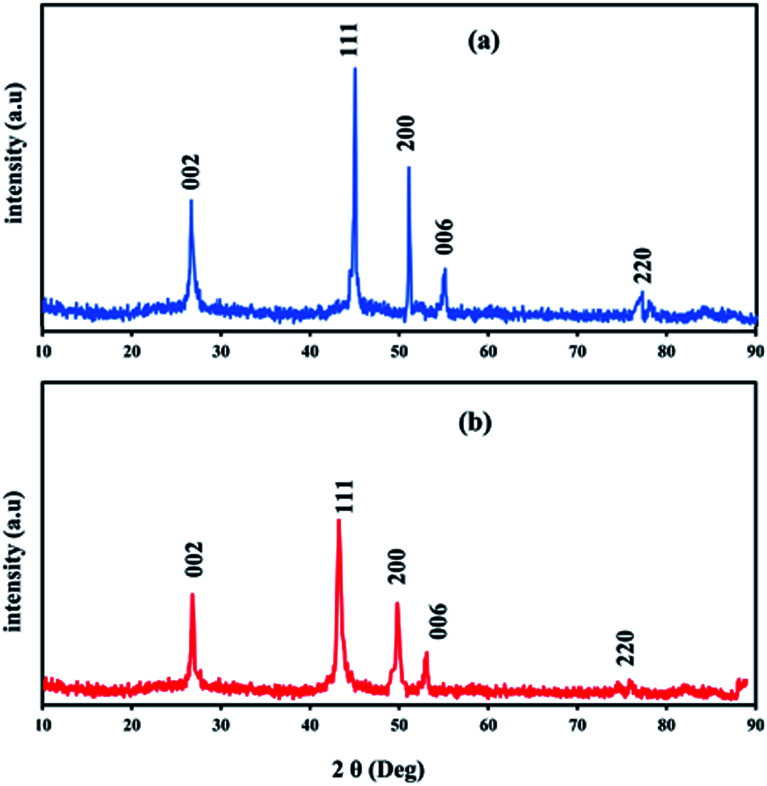
XRD patterns of (a) Ni and (b) NiCo nanoparticles electrodeposited on/in the ErN-GO/CCE.

Fig. 4 shows typical XRD patterns of Ni and NiCo alloy nanoparticles deposited on/in the ErN-GO/CCE. As shown in Fig. 4a (Ni/ErN-GO/CCE), the diffraction peaks at 2*θ* values of 44.40°, 51.65° and 76.75° related to the Ni planes of (111), (200) and (220) respectively, which correspond to face-centered cubic (fcc) structure of Ni.^49^ For NiCo/ErN-GO/CCE, XRD pattern (Fig. 4b) is similar with that of the alone Ni nanoparticles, indicating that no remarkable phase change detected with the incorporation of Co^2+^ ions. While, the peak of Ni plane (111) and also other peaks in the NiCo nanoparticles shift to the lower angle region and becomes broader than alone Ni nanoparticles. The addition of Co causes the shift of XRD patterns to lower angel values that cause increasing lattice parameter, expand the lattice parameter and the interplanar crystal spacing. Ni and Co are slightly different in the cell parameters, which the peak located at (200) of the Ni and Co corresponds to 51.5° and 51.9°, respectively. On the other hand, as can be seen from Fig. 4b, there is only a single peak between 51° to 52°, which indicates the formation of NiCo alloy.^50^ Clear inspection of both patterns shows that there are no additional peaks related to Ni, Co or mixed oxides that have poor electroconductivity and electrocatalysis activities in compared with metal alloys.^51^ The peak at 55° (006) corresponds to the supporting material (carbon-ceramic). The peak at 27° (002) corresponds to graphite-like carbon. The average crystallite size of NiCo nanoparticles was calculated using the Scherrer formula from peak located at 44.40° according to the below equation:
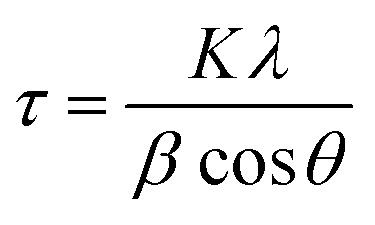
where *τ* is the mean size of the crystal, *K* is a dimensionless shape factor, with a value close to 1, *λ* is the X-ray wavelength (0.15406 nm), *β* is the line broadening at half the maximum intensity (FWHM) and *θ* is the Bragg angle.^52^ The calculated average size of electrodeposited NiCo nanoparticles on the ErN-GO/CCE according to the diffraction peak of Ni (111) is collected as 23.25 nm which is in good agreement with the result of FESEM.

### 3.2. Electrochemical characterization

**Fig. 5:**
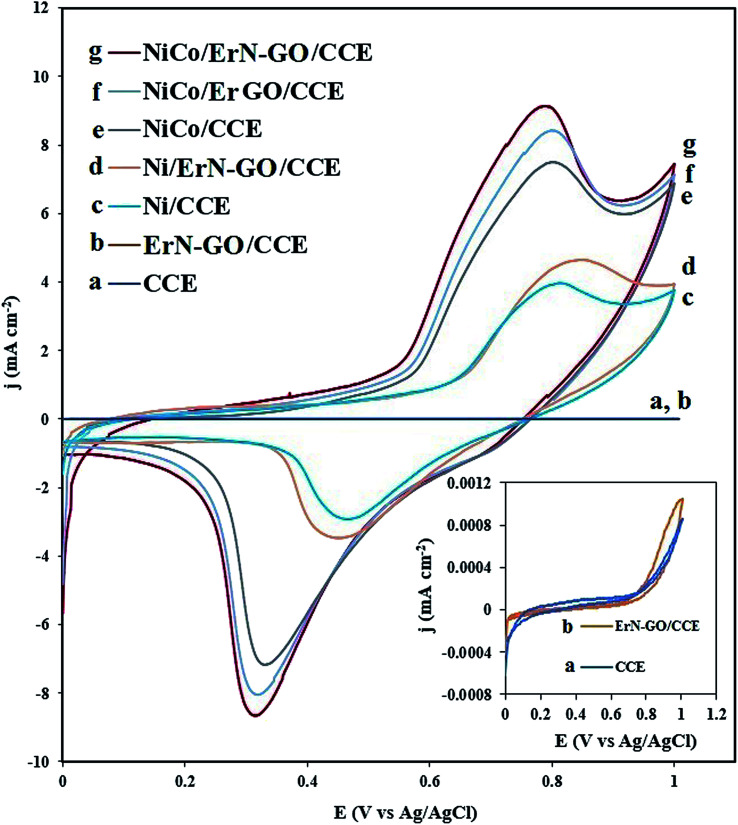
CVs of the (a) CCE, (b) ErN-GO/CCE, (c) Ni/CCE, (d) Ni/ErN-GO/CCE, (e) NiCo/CCE, (f) NiCo/ErGO/CCE and (g) NiCo/ErN-GO/CCE in 0.1 M KOH solution at 50 mV s^−1^. Inset is the CVs of (a) CCE and (b) ErN-GO/CCE in different scale.

Fig. 5 shows the cyclic voltammograms (CVs) of bare CCE (a and inset a), ErN-GO/CCE (b and inset b), Ni/CCE (c), Ni/ErN-GO/CCE (d), NiCo/CCE (e), NiCo/ErGO/CCE (f) and NiCo/ErN-GO/CCE (g) in 0.1 M KOH solution at a scanning rate of 50 mV s^−1^. As shown in inset of Fig. 5, there were no reaction peak current at the bare CCE (curve a) and ErN-GO/CCE (curve b) in the studied potential ranges. However, a pair of distinguished redox peaks observed for the Ni and NiCo nanoparticles modified electrodes at the Ni/CCE (curve c), Ni/ErN-GO/CCE (curve d), NiCo/CCE (curve e), NiCo/ErGO/CCE (curve f) and NiCo/ErN-GO/CCE (curve g), which are related to the Ni^2+^/Ni^3+^ redox couple. The NiCo/ErN-GO/CCE presents much higher anodic and cathodic current density. The changes observed in the redox peak currents and potentials are extremely related to the phase transformation of Ni(OH)_2_. NiCo alloy nanoparticles can remarkably decrease the formation of the inefficient γ-NiOOH species and on the other hand stabilize the β-NiOOH form in the alkaline media.^53^ It is well known that the β-NiOOH phase is defined as a better electroactive component for high electrochemical performance in alkaline media.^54^ These results implied that the presence of Co^2+^ in the structure of the NiCo/ErN-GO/CCE has been extremely improved the electrochemical performance of the bimetallic NiCo alloy nanoparticles in comparison with alone Ni.^55^ Additionally, comparison of the CV of NiCo/ErN-GO/CCE with NiCo/CCE shows that the NiCo/ErN-GO/CCE has higher anodic and cathodic current density, which suggests the ErN-GOs have more accessible surface area and active sites for electrodeposition of NiCo. Therefore, it can be seen that, in contrast to the CCE substrate, the ErN-GO/CCE and ErGO/CCE have large specific surface area and favorable nano-structure, which have significant effect on the both anodic and cathodic current density.^56^ Also, the ErN-GO has better performance than the ErGO due to the presence of N atoms in the structure of the ErGO. The resulted improvement in the electrochemical performance of NiCo/ErN-GO/CCE can be attributed to the synergistic effect of alloy structure and ErN-GO/CCE substrate. Moreover, due to the influence of the NiCo alloy structure, the peak potential of Ni(ii)/Ni(iii) shifted to the negative direction.^55^ This phenomenon is in accordance with the higher specific area of the NiCo alloy nanoparticles, which increases the number of active sites on the surface of NiCo alloy nanoparticles at the interface of electrode and electrolyte. On the other hand, the NiCo/ErN-GO/CCE has much more uniform NiCo alloy nanoparticles due to the synergistic effect from ErN-GO under the same loading amount, thus the NiCo/ErN-GO/CCE shows the largest anodic and cathodic current density.^34^

**Fig. 6:**
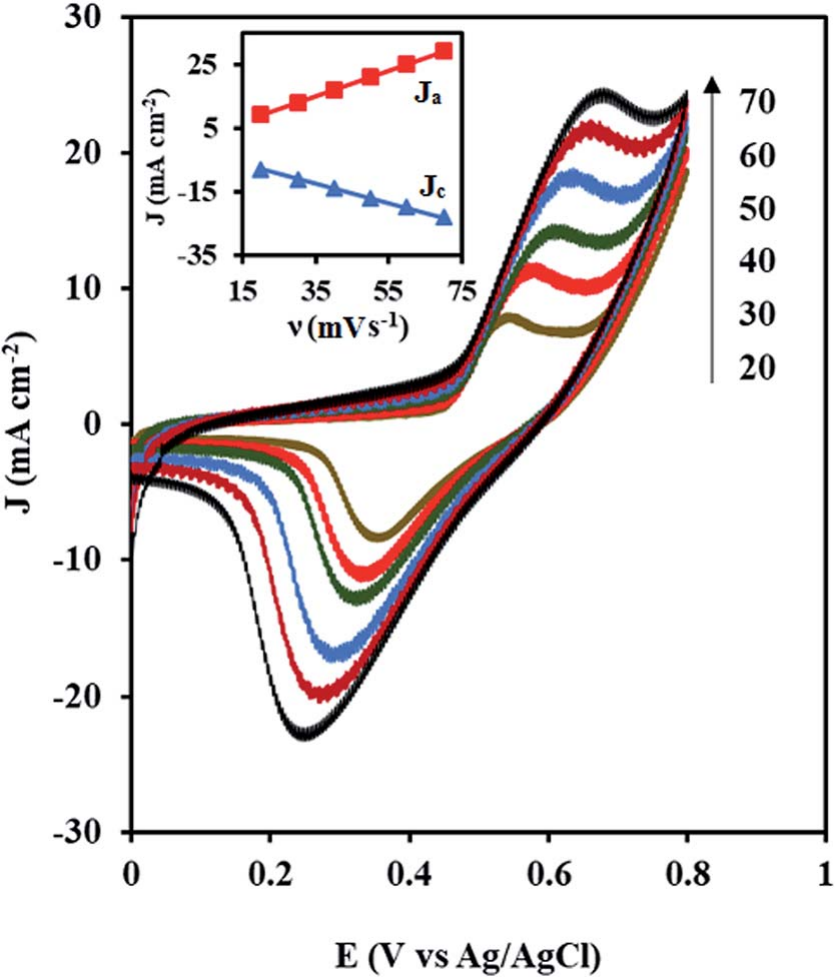
CVs of the NiCo/ErN-GO/CCE at different scan rates (20, 30, 40, 50, 60 and 70 mV s^−1^) in 0.1 M KOH at 25 °C. Inset is the anodic (*J*_a_) and cathodic (*J*_c_) peak current densities with the scan rate (*ν*).

Fig. 6 shows the CVs of the NiCo/ErN-GO/CCE recorded in 0.1 M KOH solution at different scan rates. As can be seen, both anodic and cathodic peak currents density increased clearly with increasing potential scan rates. Additionally, by increasing the scan rate, the anodic peak current density shows a potential shift toward positive values, while the cathodic peak current density is shifted toward negative potential values. A plot of the peak current density, *J*_p_ for both anodic (*J*_a_) and cathodic (*J*_c_) peaks with the scan rate (*ν*) is also shown in the inset of Fig. 6. As can be seen in the inset of Fig. 6, both peak currents density (*J*_a_ and *J*_c_) are linearly proportional to the scan rate, which indicates that this reaction is surface-confined.^57^

### 3.3. Electrocatalytic activity towards methanol and ethanol oxidation

**Fig. 7:**
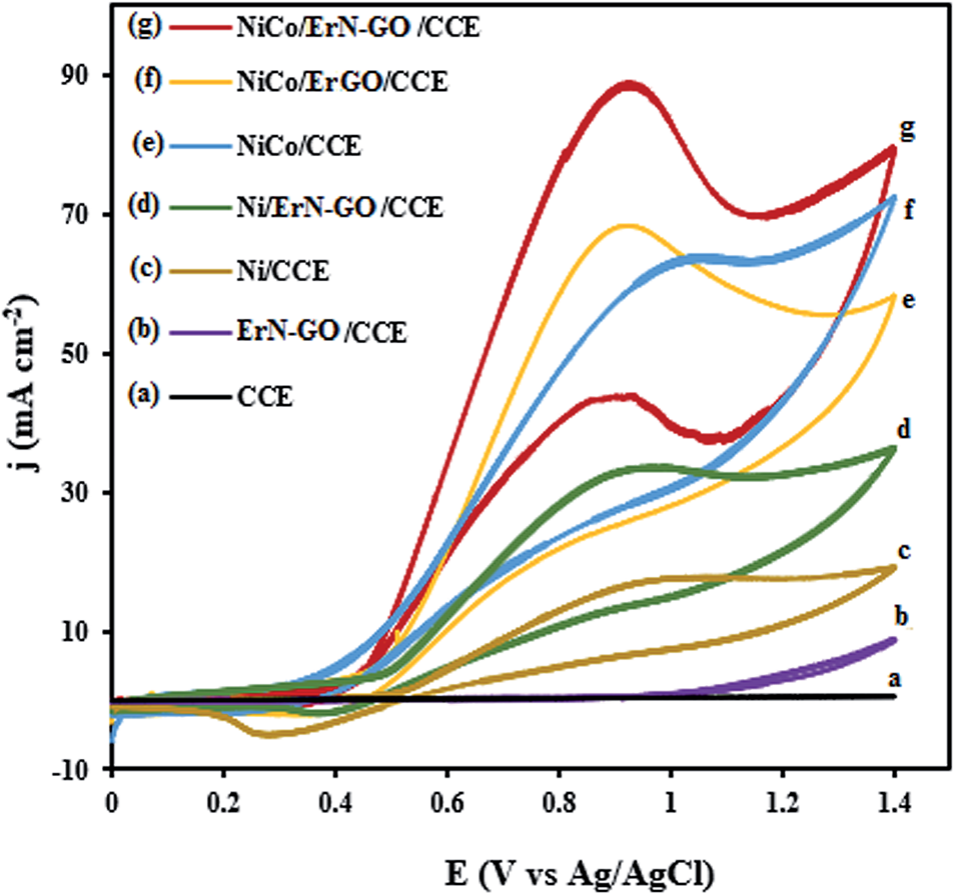
CVs of the (a) CCE, (b) ErN-GO/CCE, (c) Ni/CCE, (d) Ni/ErN-GO/CCE, (e) NiCo/CCE, (f) NiCo/ErN-GO/CCE and (g) NiCo/ErN-GO/CCE in 0.1 M KOH + 0.1 M methanol at 50 mV s^−1^.

Fig. 7 represents the CVs of the bare CCE (a), ErN-GO/CCE (b), Ni/CCE (c), Ni/ErN-GO/CCE (d), NiCo/CCE (e), NiCo/ErGO/CCE (f) and NiCo/ErN-GO/CCE (g) in 0.1 M KOH + 0.1 M methanol at 50 mV s^−1^. As can be seen in Fig. 7, in curves a and b, there is no detectable signals for methanol electrooxidation on the bare CCE and N-ErGO/CCE, which indicate that CCE and N-ErGO/CCE are inactive and clearly show that these electrodes exhibit no electrocatalytic activity towards methanol oxidation without metal loading. However, NiCo alloy and Ni nanoparticles electrodeposited electrodes (curves d–g) show electrocatalytic activity towards methanol oxidation. For the NiCo/ErN-GO/CCE, the electrooxidation peak current density increased with the presence of Co atoms as the alloy electrocatalyst from 38.20 mA cm ^−2^ for the Ni/ErN-GO/CCE to the 88.04 mA cm^−2^ and also increased by the presence ErN-GO as electrocatalyst support from 62.15 mA cm^−2^ for the NiCo/CCE to the 88.04 mA cm^−2^ at the NiCo/ErN-GO/CCE. Similarly, the peak current density for the NiCo/ErN-GO/CCE increased by N doping of ErGO from 71.06 mA cm^−2^ for the NiCo/ErGO/CCE to the 88.04 mA cm^−2^ NiCo/ErN-GO/CCE. On the other hand, for the NiCo/ErN-GO/CCE, the methanol oxidation reaction was sharply affected by the presence of Co atoms and N doped ErGO in the structure of nanocomposite and consequently methanol electrooxidation current density was significantly increased. In the field of fuels electrooxidation, the onset potentials considered as an important parameter for determining the electrocatalytic activity, and the more negative onset potential, indicates better performance, higher electrocatalytic activity and the lower overpotential.^58^ In addition, the onset potential of methanol electrooxidation shifts to a lower value on NiCo/ErN-GO/CCE than on others electrocatalysts. It is clear that the existing of Co atoms alongside Ni nanoparticles dramatically increases the current density and decreases the onset potential. The co-existing in the electrocatalyst composition modifies both, the peak current and the onset potential. This change in the *j*–*E* profile is likely due to the formation of CoOOH from the corresponding cobalt hydroxide, Co(OH)_2_, which starts before NiOOH formation.^59^ Also, ErN-GO/CCE with flaky structure and small dimension can provide a larger surface area of the modified electrode and effectively improves the electroactivity of the NiCo/ErN-GO/CCE for methanol oxidation. Additionally, ErN-GO/CCE would facilitates the electron transfer of methanol electrooxidation between the interface of the electrocatalyst and solution^60^ due to the introduction of heteroatoms such as N into graphene skeleton can break its electroneutrality and effectively engineer its electronic structure, thus improving the reactivity when compared to their undoped analogs.^61^ Furthermore, in the backward scan, a cathodic peak is observed at 0.35 V *vs.* Ag/AgCl related to the NiOOH species. As mentioned above, the electrochemically active species in Ni based modified electrodes is NiOOH, so we should expect that the methanol electrooxidation starts with the NiOOH surface formation.^62–65^ The current density of the cathodic peak in reverse scan is considerably less intense because of NiOOH consumption during the methanol oxidation reaction.^66^

**Fig. 8:**
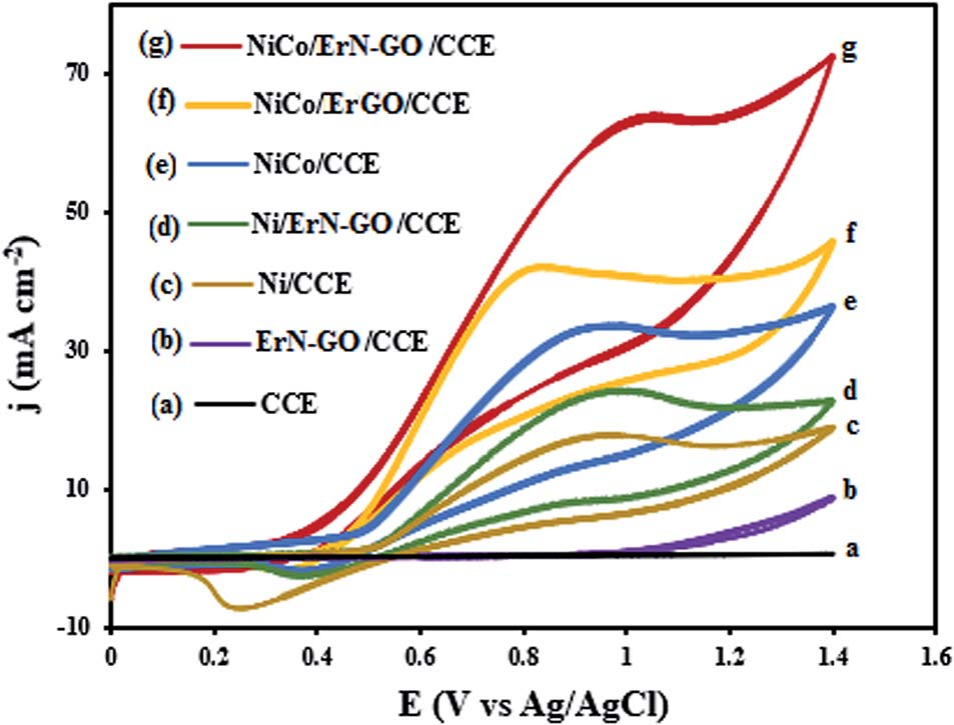
CVs of the (a) CCE, (b) ErN-GO/CCE, (c) Ni/CCE, (d) Ni/ErN-GO/CCE, (e) NiCo/CCE, (f) NiCo/ErN-GO/CCE and (g) NiCo/ErN-GO/CCE in 0.1 M KOH + 0.1 M ethanol at 50 mV s^−1^.

CVs for the oxidation of ethanol at the CCE (a), ErN-GO/CCE (b), Ni/CCE (c), Ni/ErN-GO/CCE (d), NiCo/CCE (e), NiCo/ErGO/CCE (f) and NiCo/ErN-GO/CCE (g) in 0.1 M KOH + 0.1 M ethanol at a scan rate of 50 mV s^−1^ are shown in Fig. 8. As in the case of methanol, CCE (a) and ErN-GO/CCE (b) are inactive and exhibit no electrocatalytic activity towards ethanol oxidation. While other modified electrodes show a typical ethanol oxidation current peak in the forward scan. For the NiCo/ErN-GO/CCE, the anodic peak current density is the highest and the onset potential is negativist, which suggests that the NiCo/ErN-GO/CCE has the positive effects on promoting the oxidation of ethanol by lowering its overpotential. The lower onset oxidation potential and highest anodic peak current density for NiCo alloy nanoparticles NiCo/ErN-GO/CCE, disclose that the NiCo alloy has the best electrocatalytic activity towards ethanol oxidation. Therefore, as mentioned above, due to the higher surface concentration of the β-NiOOH form in the Ni, NiCo nanoparticles in comparison to alone Ni nanoparticles because of the presence of the Co, the alloy modified electrodes generate a higher electrocatalytic activity towards methanol and ethanol electrooxidation in KOH solution.^67^ Moreover, some part of this increased activity is attributed to the extraordinary properties of GO, such as the extended π conjugation between the C atoms and the strong interaction between GO and metal nanoparticles.^68^ Additionally, GO has outstanding conductivity and excellent mechanical properties, which help enhance the activity of the electrocatalyst for alcohol oxidation. However, the presence of N atoms and produced N functional groups on the GO generated additional anchor sites for metal seeding and deposition, which led to improved NiCo nanoparticle size, uniformity, and dispersion.^34,69,70^ The electrocatalytic performance of the prepared electrocatalysts is measured and listed in Table 1.

**Table 1:** Electrocatalytic activity and kinetic parameters of the prepared electrodes for methanol and ethanol oxidation

Fuel	Electrodes	*E*_onset_ (V)	*E*_pf_ (V)	*J*_pf_ (mA cm^−2^)	*α*	*J*_0_ (mA cm^−2^)
Methanol	NiCo/ErN-GO/CCE	0.44	0.88	88.04	0.44	1.89
	NiCo/ErGO/CCE	0.46	0.89	71.06	0.41	1.74
	NiCo/CCE	0.47	0.91	62.15	0.39	1.65
	Ni/ErN-GO/CCE	0.49	0.91	38.20	0.36	1.42
	Ni/CCE	0.50	0.97	21.51	0.31	1.29
	ErN-GO/CCE	0	0	0	0	0
	CCE	0	0	0	0	0
Ethanol	NiCo/ErN-GO/CCE	0.38	0.81	64.23	0.48	1.31
	NiCo/ErGO/CCE	0.42	0.79	40.56	0.44	1.26
	NiCo/CCE	0.44	0.84	34.25	0.41	1.18
	Ni/ErN-GO/CCE	0.52	0.89	22.31	0.38	0.97
	Ni/CCE	0.55	0.91	17.53	0.35	0.81
	ErN-GO/CCE	0	0	0	0	0
	CCE	0	0	0	0	0

#### 3.3.1. Kinetic investigation of electrooxidation of methanol and ethanol

**Fig. 9:**
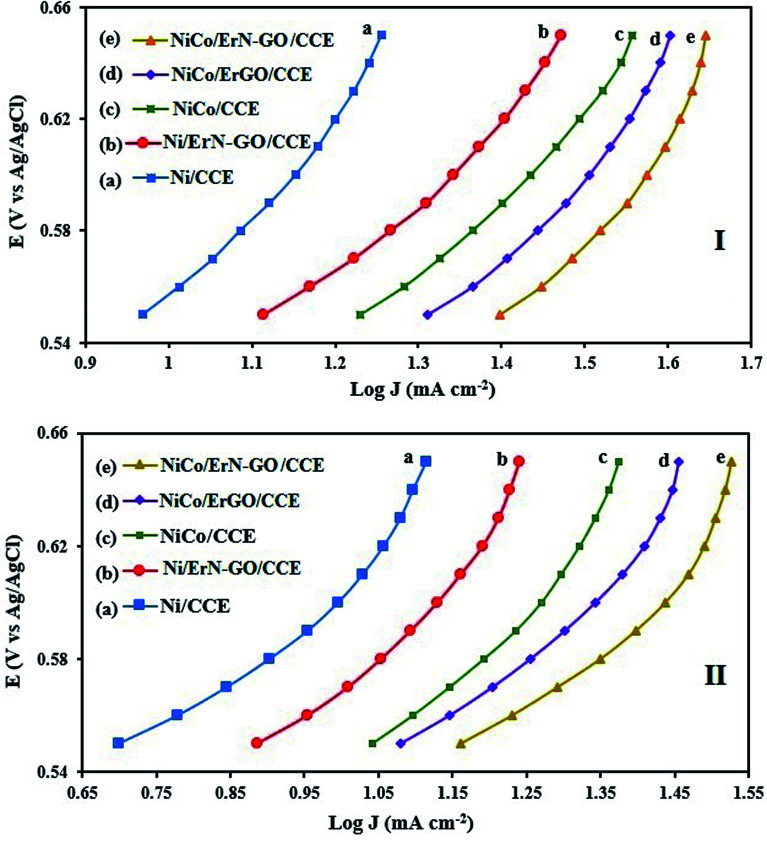
(I) Tafel plots of the (a) Ni/CCE, (b) Ni/ErN-GO/CCE, (c) NiCo/CCE, (d) NiCo/ErGO/CCE and (e) NiCo/ErN-GO/CCE for methanol electrooxidation and (II) Tafel plots of the (a) Ni/CCE, (b) Ni/ErN-GO/CCE, (c) NiCo/CCE, (d) NiCo/ErGO/CCE and (e) NiCo/ErN-GO/CCE for ethanol electrooxidation.

To investigate the kinetic parameters for methanol and ethanol electrooxidation at the Ni/CCE (a), Ni/ErN-GO/CCE (b), NiCo/CCE (c), NiCo/ErGO/CCE (d) and NiCo/ErN-GO/CCE (e) electrocatalysts, the polarization curves in a very low scan rate (10 mV s^−1^) of mentioned electrocatalysts in 0.1 M KOH and in presence of methanol and ethanol are replotted according to *E* (*vs.* Ag/AgCl) *vs.* log *j*, as shown in Fig. 9I for methanol and Fig. 9II for ethanol, which is referred to as the Tafel plot and according to the following equation:^71^
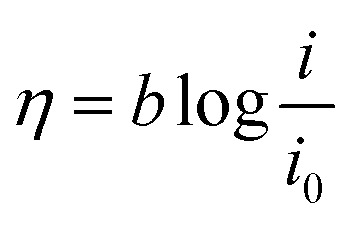
where 
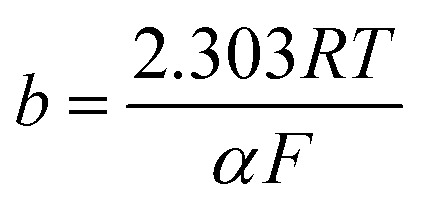
In this equation the constants *R* and *F* devoted to the universal gas constant and the Faraday constant, respectively; *T* is the temperature (in K); *α* is the charge transfer coefficient of the reaction, and *i*_0_ is the exchange current density. The value of *b*, the Tafel slope, is generated from the linear part of the Tafel region fitted with a straight line in Fig. 9I and II. The Tafel slopes for Ni/CCE (a), Ni/ErN-GO/CCE (b), NiCo/CCE (c), NiCo/ErGO/CCE (d) and NiCo/ErN-GO/CCE (e) are 214, 182, 161, 134, and 128 mV dec^−1^, for methanol electrooxidation [Fig. 9I] respectively and also 221, 198, 174, 155 and 136 mV dec^−1^ for ethanol electrooxidation [Fig. 9II] respectively. From comparing of the Tafel slopes of methanol and ethanol at the Ni/CCE (a), Ni/ErN-GO/CCE (b), NiCo/CCE (c), NiCo/ErGO/CCE (d) and NiCo/ErN-GO/CCE (e) (Fig. 9I and II), it is clear that the NiCo/ErN-GO/CCE has a lower Tafel slope, resulting in less overpotential and therefore more electrocatalytic activity. The kinetic parameters from the experimental results in Fig. 9 are also summarized in Table 1. A higher exchange current density (*j*_0_) for electrochemical reactions implies a faster electrode reaction, which is an important parameter for effective electrocatalysts and good methanol and ethanol electrooxidation performance. The value of the charge transfer coefficient *α* is affected by the nature of the modified electrocatalysts thus, this indicates that the NiCo/ErN-GO/CCE is a sufficient electrocatalyst to improve the methanol and ethanol electrooxidation reactions and has higher kinetics in comparison to others.

#### 3.3.2. Effects of potential scan rates on the electrooxidation of methanol and ethanol

**Fig. 10:**
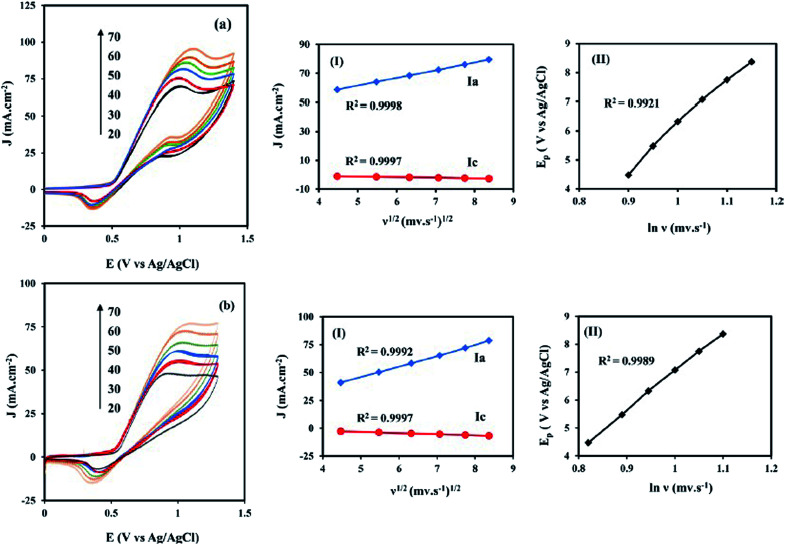
(a) CVs of the NiCo/ErN-GO/CCE at different scan rates in 0.1 M KOH + 0.1 M methanol and (b) CVs of the same electrocatalyst at different scan rates in 0.1 M KOH + 0.1 M ethanol at 50 mV s^−1^. Insets (I) are the plot of anodic peak current densities (*J*_a_) *vs. ν*^1/2^ and insets (II) are the plot of peak potential (*E*_pf_) *vs.* ln(*ν*).

For the investigation of the transport characteristics of the methanol and ethanol electrooxidation on the NiCo/ErN-GO/CCE, the effect of the scan rate (*ν*) on the electrooxidation of methanol (Fig. 10a) and ethanol (Fig. 10b) was investigated. It can be seen that the peak current density of methanol and ethanol electrooxidation increases with increasing the scan rate. The anodic peak current densities (*J*_a_) linearly proportional to *ν*^1/2^ [insets (I) of Fig. 10a and b] which implies that the electrooxidation of the methanol and ethanol on the NiCo/ErN-GO/CCE is controlled by the diffusion of methanol and ethanol. As can be seen, insets (II) of Fig. 10a and b, the peak potential (*E*_pf_) (in the forward scan), increases with the increasing of *ν*, and a linear relationship was obtained between *E*_pf_ and ln(*ν*). It is obvious that the peak potential of methanol and ethanol electrooxidation shifts to more positive values with the scan rate that may indicate the kinetic limitation of the methanol and ethanol electrocatalytic oxidation on the NiCo/ErN-GO/CCE. These results show that the electrooxidation of methanol and ethanol at the NiCo/ErN-GO/CCE are irreversible electrode processes.^72^

#### 3.3.3. Effects of methanol and ethanol concentration in electrooxidation of methanol and ethanol

**Fig. 11:**
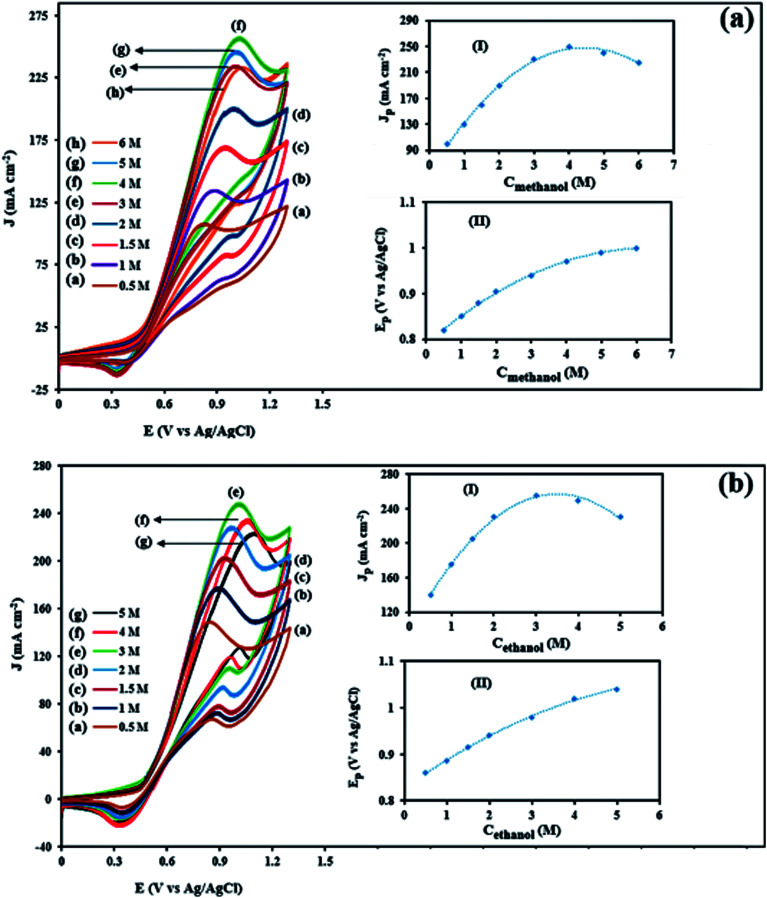
CVs of the NiCo/ErN-GO/CCE in 0.1 M KOH solution in the presence of different methanol concentrations (a) and 0.1 M KOH solution in the presence of different ethanol concentrations (b). Inset (I) and (II) show dependency of the peak potential and anodic peak current densities on the concentration of methanol and ethanol in solution, respectively.

Methanol and ethanol concentrations extremely affect the anodic current density values and play the important role in the activity of electrocatalysts. Therefore, appropriate methanol and ethanol concentration can make the fuel cell to get the best performance. Fig. 11a and b show the influence of methanol and ethanol concentration (0.5–6 M) on the corresponding CVs current densities for the NiCo/ErN-GO/CCE at a scan rate of 50 mV s^−1^. The figures show that there is a limiting correlation between the peak current density of methanol and ethanol electrooxidation and their concentration. As the methanol and ethanol concentrations gradually increased from 0.5 M to 5 M and 0.5 M to 6 M, respectively, the peak current density resulting from the electrooxidation of methanol and ethanol increased significantly and reaches the maximum of 262 mA cm^−2^ at 4 M (inset I of Fig. 11a) and 251 mA cm^−2^ at 3 M (inset I of Fig. 11b) for methanol and ethanol electrooxidation, respectively. As the methanol and ethanol concentration continues to increase, the oxidation peak current density shows a decreasing trend. The decrease of anodic peak current density may be due to that when the methanol and ethanol concentration is higher, a large number of active sites on the electrocatalyst surface are occupied by oxidation products of methanol and ethanol, which increase the poising extent of electrocatalyst and hinders the diffusion of reactant into the electrocatalyst surface.^73^ However, at higher fuel concentrations, the peaks current tends to have different mode indicating a limitation in the kinetics of the methanol and ethanol electrooxidation.^74^ Additionally, the peak potential (*E*_pf_) (in the forward scan) shift to more positive values with increasing the methanol (inset II of Fig. 11a) and ethanol (inset II of Fig. 11a) concentrations. This may be attributed to the poisoning of electrocatalyst surface and limitation in the kinetics of the methanol and ethanol electrooxidation when increasing their concentrations and the oxidative removal of the adsorbed intermediates that would shift to a more positive potential.^75^

#### 3.3.4. EIS performances

**Fig. 12:**
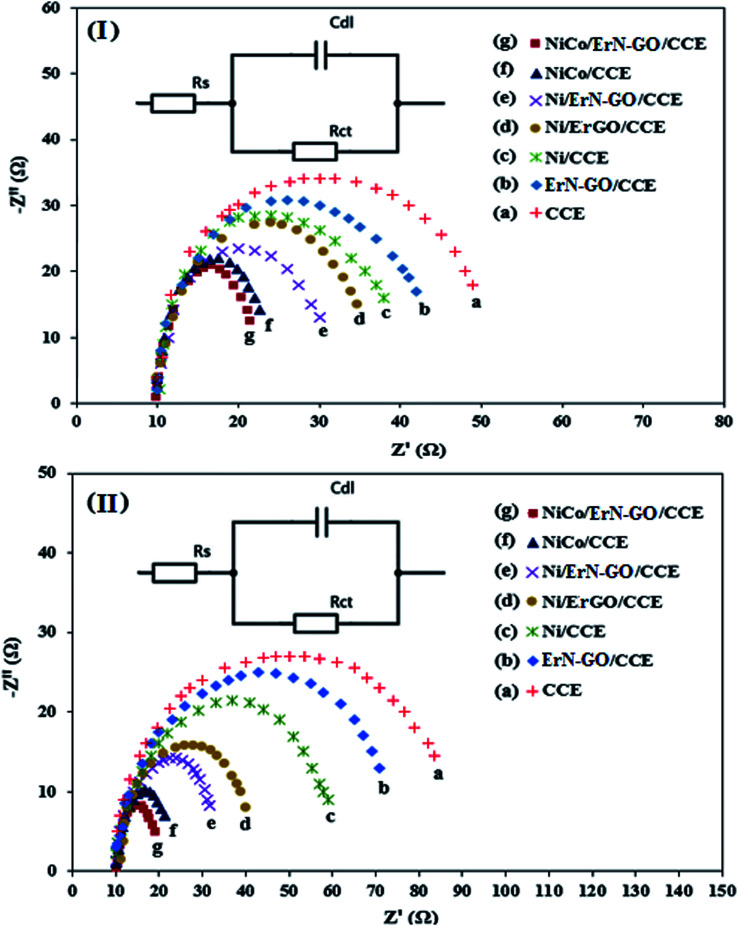
Nyquist plots of impedance measurements (at 0.6 V *vs.* Ag/AgCl) in 0.1 M KOH + 0.1 M methanol solution (I) and 0.1 M KOH + 0.1 M ethanol solution (II) on the (a) CCE, (b) ErN-GO/CCE, (c) Ni/CCE, (d) Ni/ErGO/CCE, (e) Ni/ErN-GO/CCE, (f) NiCo/CCE and (g) NiCo/ErN-GO/CCE. Insets are the Randles equivalent circuit for NiCo/ErN-GO/CCE.

The electrochemical resistance of the prepared electrocatalysts is an important parameter affecting their performance in DAFCs. The electrochemical impedance spectroscopy (EIS) technique is a powerful electrochemical technique can be used to investigate the validity of the electrocatalytic process and studying the conductivity and ion transport/diffusion ability at the interface between the surface of the modified electrode and the electrolyte.^77^Fig. 12I and II illustrate the Nyquist diagrams of the various prepared electrocatalysts including CCE (a), N-ErGO/CCE (b), Ni/CCE (c), Ni/ErN-GO/CCE (d), NiCo/CCE (e), NiCo/ErGO/CCE (f) and NiCo/ErN-GO/CCE (g) recorded in the frequency range of 0.1 Hz to 10 kHz under amplitude of 10 mV and constant bias potential 0.6 V in 0.1 M KOH containing 0.1 M methanol [Fig. 12I] and 0.1 M KOH containing 0.1 M ethanol [Fig. 12II]. As shown in the Fig. 12I and II, the semicircle diameter for NiCo/ErN-GO/CCE is smaller than the other electrocatalysts, indicating the lower charge transfer resistance of methanol and ethanol electrooxidation reaction and considerably enhanced kinetics on the surface of the NiCo/ErN-GO/CCE. Smaller charge transfer resistance suggested a faster electron transfer during methanol and ethanol electrooxidation reaction. The above-mentioned results indicate that the electron conductivity and ions transport of NiCo/ErN-GO/CCE is much better than the other prepared electrocatalysts. This improvement can be attributed to the presence of NiCo alloy nanoparticles in a higher surface area. Moreover, the presence of ErN-GO/CCE, as a high conductive material, accelerates the electron transfer process.^78^ Finally, the insets of Fig. 12 show the experimental data that are fitted to standard Randles equivalent circuits for NiCo/ErN-GO/CCE surface analysis for methanol (inset of Fig. 12I) and ethanol (inset of Fig. 12II) electrooxidation, which comprises the solution resistance (*R*_s_), the charge transfer resistance (*R*_ct_) and the constant phase element for the cases of NiCo/ErN-GO/CCE.

#### 3.3.5. Long-term stability performances

**Fig. 13:**
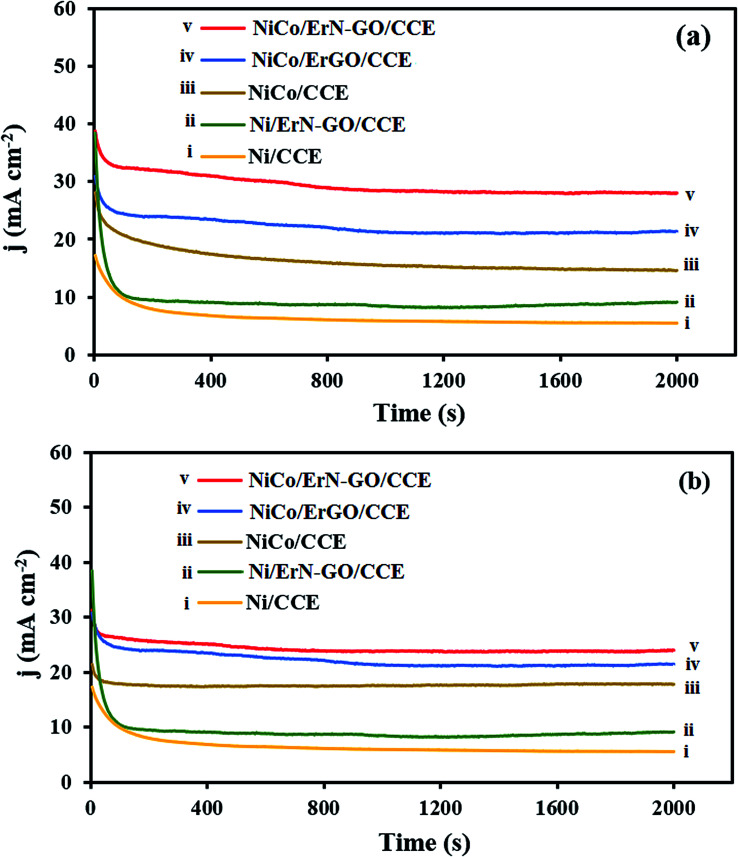
(a) Chronoamperometric curves of the (i) Ni/CCE, (ii) Ni/ErN-GO/CCE, (iii) NiCo/CCE, (iv) NiCo/ErGO/CCE and (v) NiCo/ErN-GO/CCE for 0.1 M methanol electrooxidation and (b) Chronoamperometric curves of the (i) Ni/CCE, (ii) Ni/ErN-GO/CCE, (iii) NiCo/CCE, (iv) NiCo/ErGO/CCE and (v) NiCo/ErN-GO/CCE for 0.1 M ethanol electrooxidation.

From practical view, besides the required high electrocatalytic activity for an electrocatalyst, long-term stability of the prepared electrocatalyst is also important for their real applications in DAFCs. By using the chronoamperometry technique, the electrocatalytic stability of the prepared electrocatalysts for electrooxidation of methanol and ethanol at a constant potential step (0.6 V) for a period of time (2000 s) was investigated in alkaline media [Fig. 13a (for methanol at the (i) Ni/CCE, (ii) Ni/ErN-GO/CCE, (iii) NiCo/CCE, (iv) NiCo/ErGO/CCE and (v) NiCo/ErN-GO/CCE) and (b) (for ethanol at the (i) Ni/CCE, (ii) Ni/ErN-GO/CCE, (iii) NiCo/CCE, (iv) NiCo/ErGO/CCE and (v) NiCo/ErN-GO/CCE)]. As shown in Fig. 13a and b, at the initial step of electrolysis, the current density of methanol and ethanol electrooxidation reduces rapidly, and afterward presents a relatively stable current density with time. The rapid decrease at the initial stage might be due to the double layer charging process and the rapid consumption of methanol and ethanol close to the interface between electrocatalyst and solution.^76^ After that, the molecules of the fuel reach the electrocatalyst surface at a constant rate, and the current density reaches a constant value and no change in the current density is observed for electrooxidation. On the other hand, it can be observed that the oxidation current density at the NiCo/ErN-GO/CCE is the highest among the all prepared electrocatalysts. This could be due to the high activity of NiCo alloy nanoparticles and higher surface area and high conductivity of the supporting material (ErN-GO/CCE).

Finally, we compare the results of methanol (MeOH) and ethanol (EtOH) electrooxidation activity of this work to response of some other earlier electrocatalysts reported in the literature (Table 2). The data in Table 2 show that the NiCo/ErN-GO/CCE exhibits better or comparable electrocatalytic performance than other reported electrocatalysts towards methanol and ethanol electrooxidation. This improved performance could be contributed to the unique morphology of the prepared ultrathin ErN-GO nanosheets on the surface of CCE and enhancement of the electrochemically active surface area of NiCo alloy nanoparticles that provide more electrochemical active sites for the methanol and ethanol electrooxidation.

**Table 2:** Comparison of different electrocatalysts for methanol and ethanol electrooxidation

Electrocatalyst	Compositions of electrolyte solution	*E*_onset_ (V)	*E*_pf_ (V)	*J*_pf_ (mA cm^−2^)	Ref
Ni–Co/RGO/CPE	1.0 M KOH + 1.0 M EtOH	0.38	0.75	22.21	32
Pd/N-P-G	1 M KOH + 0.1 M MeOH	−0.41	−0.20	108.6	34
Ni_3_Co_1_/C-N/CNT	1 M KOH + 0.1 M MeOH	0.36	0.80	208	50
	1.0 M KOH + 1.0 M EtOH	0.38	0.80	180	
Ni_0.2_Co_0.2_/CNF	1 M KOH + 3 M EtOH	0.36	0.71	105	79
FePt/GCE	0.5 M H_2_SO_4_ + 0.5 M MeOH	0.37	0.78	2.85	80
PtFe/Au/ITO	0.5 M KOH + 0.5 M MeOH	−0.25	0.2	59	81
NiPt	1 M KOH + 0.1 M MeOH	−0.50	0.0	10.12	82
Ni/PtRu	1.0 M NaOH + 1.0 M EtOH	−0.51	−0.20	45	83
PtRu/SnO_2_/CNT	0.5 M H_2_SO_4_ + 1 M MeOH	0.42	0.82	55	84
PdNiCeO	1.0 M KOH + 1.0 M EtOH	−0.30	0.15	5.4	85
Ni@Pt/CCE	0.5 M NaOH + 0.5 M EtOH	−0.62	−0.21	7.76	86
NiO-MOF/rGO	1 M NaOH + 3 M MeOH	0.41	0.83	174	87
PtNi/ERPGO	0.5 M H_2_SO_4_ + 0.5 M MeOH	0.40	0.60	4.5	88
NiCo_2_O_4_/rGO	1 M KOH + 0.5 MeOH	0.25	0.37	3.22	89
NiCo/ErN-GO/CCE	0.1 M KOH + 0.1 M MeOH	0.38	0.80	88.04	This work
	0.1 M KOH + 0.1 M EtOH	0.36	0.81	64.23	

## 4. Conclusions

A simple electrochemical method was used to fabricate electrochemical reduced nitrogen-doped graphene oxide (ErN-GO) on the carbon-ceramic electrode as substrate for the preparation of a non-platinum electrocatalyst. NiCo alloy nanoparticles were electrodeposited by an electrochemical route and characterized by different techniques including field emission scanning electron microscopy (FESEM), X-ray diffraction (XRD), and EDX and map analysis. The FTIR spectroscopy and EDX analyses confirm the presence of the nitrogen atoms in ErN-GO. The prepared electrocatalysts; NiCo and alone Ni nanoparticles electrodeposited on different substrates, were characterized by cyclic voltammetry, chronoamperometry and EIS methods and obtained results showed that the NiCo/ErN-GO/CCE has the highest electrocatalytic activity for methanol (*J*_p_ = 88.04 mA cm^−2^) and ethanol (*J*_p_ = 64.23 mA cm^−2^) electrooxidation in alkaline media. Based on the electrocatalytic activity, stability, simple preparation method and lower price of present electrocatalyst, it is expected that this material can be used as promising anode materials applied to methanol and ethanol oxidation in DAFCs.

## Conflicts of interest

There are no conflicts to declare.

## Supplementary Material
